# COVID-19 and the Kidney: From Epidemiology to Clinical Practice

**DOI:** 10.3390/jcm9082506

**Published:** 2020-08-04

**Authors:** Ida Gagliardi, Gemma Patella, Ashour Michael, Raffaele Serra, Michele Provenzano, Michele Andreucci

**Affiliations:** 1 Renal Unit, Department of Health Sciences, Magna Graecia University, 88100 Catanzaro, Italy; ida_88@libero.it (I.G.); gemmapatella@hotmail.it (G.P.); ashourmichael@yahoo.com (A.M.); michiprov@hotmail.it (M.P.); 2 Interuniversity Center of Phlebolymphology (CIFL), International Research and Educational Program in Clinical and Experimental Biotechnology, Headquarters, Magna Graecia University, 88100 Catanzaro, Italy; rserra@unicz.it

**Keywords:** SARS-CoV-2, acute kidney injury, dialysis, renal transplantation, pandemic

## Abstract

The new respiratory infectious disease coronavirus disease 2019 (COVID-19) that originated in Wuhan, China, in December 2019 and caused by a new strain of zoonotic coronavirus, named severe acute respiratory syndrome-coronavirus-2 (SARS-CoV-2), to date has killed over 630,000 people and infected over 15,000,000 worldwide. Most of the deceased patients had pre-existing comorbidities; over 20% had chronic kidney disease (CKD). Furthermore, although SARS-CoV-2 infection is characterized mainly by diffuse alveolar damage and acute respiratory failure, acute kidney injury (AKI) has developed in a high percentage of cases. As AKI has been shown to be associated with worse prognosis, we believe that the impact of SARS-CoV-2 on the kidney should be investigated. This review sets out to describe the main renal aspects of SARS-CoV-2 infection and the role of the virus in the development and progression of kidney damage. In this article, attention is focused on the epidemiology, etiology and pathophysiological mechanisms of kidney damage, histopathology, clinical features in nephropathic patients (CKD, hemodialysis, peritoneal dialysis, AKI, transplantation) and prevention and containment strategies. Although there remains much more to be learned with regards to this disease, nonetheless it is our hope that this review will aid in the understanding and management of SARS-CoV-2 infection.

## 1. Introduction

By December 2019, unusual cases of pneumonia had been reported in the city of Wuhan, located in central China’s Hubei province. On January 12th 2020, the World Health Organization (WHO) stated that the disease was caused by a novel coronavirus, named severe acute respiratory syndrome-coronavirus-2 (SARS-CoV-2), belonging to the β-coronavirus cluster, which also includes the severe acute respiratory syndrome (SARS) and Middle East respiratory syndrome (MERS) viruses [1,2].

The resulting SARS-CoV-2 related disease was defined as the novel coronavirus disease 2019, or COVID-19, that rapidly spread throughout China, followed by an increasing number of cases in all continents with the exception of Antarctica, resulting in a global pandemic.

The data of the global burden of COVID-19 are impressive. Indeed, currently (available data up to 23 July 2020) the coronavirus is affecting 212 countries and territories around the world, with over 15 million cases and over 630,000 confirmed deaths (global mortality: 5.4%) [3,4].

The Americas and Europe are the most affected continents in the world. In the United States of America (U.S.A.), 4,103,674 cases and over 146,000 deaths have been reported. In Europe, many outbreaks are steadily rising in Russia, Spain, UK and Italy, with over 200,000 overall deaths in the continent. However, in China the rate of new cases has progressively decreased substantially since March [3]. The pandemic has also had a severe economic impact. In fact, to help improve the clinical management of patients infected with the virus, Europe has mobilized more than 10 million euros from research funding and the United Kingdom has also invested £20,000,000 to allow for the development of a COVID-19 vaccine [5]. It has also been estimated that in the U.S.A., the health care costs for a single infected patient is over $3000, resulting in an overall predicted expenditure of $654.0 billion over the entire period of the pandemic [6,7].A flu-like syndrome of mild severity has been observed in most cases (80%) of COVID-19 [8,9], but in 20% of cases there have been other complications such as interstitial pneumonia with a variable degree of respiratory failure [10] as well as thromboembolic complications, including venous thromboembolism (VTE), ischemic stroke and acute coronary syndrome (ACS)/myocardial infarction [11]. Although the principal features associated with COVID-19 are diffuse alveolar damage and acute respiratory failure, kidney impairment has also often developed, with the frequent onset of acute kidney injury (AKI) in patients infected by SARS-CoV-2. In addition, more than 20% of deceased patients were affected by chronic kidney disease (CKD) [11]. For this reason, the kidney’s role in COVID-19 needs to be investigated [8]. The aim of this review is to describe the main aspects of COVID-19 infection, namely the epidemiology and pathophysiology, together with prevention strategies and the contribution of kidney diseases to the prognosis of COVID-19 affected patients.

## 2. Epidemiology

Globally, over 15 million confirmed cases of COVID-19 have been reported to date (23 July 2020) [4].

Such a large number of affected patients have been caused by the way in which the virus is transmitted. COVID-19 is more contagious when compared to MERS and SARS since the virus spreads by human-to-human transmission via direct or fecal contact or droplets [7]. Furthermore, the possibility that the virus may be transmitted by asymptomatic individuals or by individuals within the incubation period, may also explain the high contagiousness of the disease [12].

COVID-19 infection is thought to have an incubation period up to 14 days following exposure, with most symptoms showing around four to five days after exposure [13]. However, case reports with longer incubation periods (up to 27 days) have also been reported [11]. An updated publication confirmed these findings, with the incubation lasting as long as 24 days (range: 0–24 days; median: 3.0 days) [7].

Nonetheless, the onset of symptoms is variable amongst infected patients, and the interval during which an individual with COVID-19 is contagious remains uncertain. It appears that SARS-CoV-2 can be transmitted prior to the development of symptoms and throughout the course of illness [14]. The infection can involve all age groups, including children, and, moreover, males suffer a disproportionately higher number of deaths than females according to data from cohorts of patients in China, Italy and the United States [15,16]. Patients admitted at the Tongji Hospital, the major endemic area, and positive for COVID-19, showed a median age of over 60-years and a large part (up to 42%) were affected by 1 or more comorbidities, including diabetes mellitus, cardiovascular disease, hypertension, chronic lung disease, cancer, chronic kidney disease, immunocompromising conditions, severe obesity (body mass index ≥ 40) and liver disease [17,18]. Similar evidence was derived from a cohort of 5700 hospitalized patients on the other side of the world, namely in the New York city area in the U.S.A. [15]. Moreover, the data of 333 children with confirmed SARS-CoV-2 from 11 case series were analyzed [19]. Intriguingly, despite the recent evidence that children have the same risk for infection than adults, few cases have been admitted to intensive care units (3%) and only a few deaths have been registered. The less-severe infection has been principally related to the fact that children have a stronger innate immune response and a minor prevalence of comorbidities (arterial hypertension, cardiovascular diseases) than adults [20].

Most patients also showed an inflammatory status and coagulopathies with elevated levels of high-sensitivity C-reactive protein and serum lactose dehydrogenase [8]. Interestingly, up to 15% of the hospitalized COVID-19 patients had at least one kidney abnormality represented by increased blood urea nitrogen or reduced estimated glomerular filtration rate (eGFR) which is the best marker of kidney function. Moreover, findings from different cohorts of hospitalized patients showed that 26–63% of patients presented proteinuria at admission or developed proteinuria during their stay in hospital, proteinuria being considered the most recognized sign of kidney damage [7,8]. An individual risk profile also found that COVID-19 patients with kidney abnormalities, compared with those with normal renal function at admission, were more likely to be males, with advanced age and with a worse coagulation profile [8]. In addition, a meta-analysis [21] has confirmed that CKD is associated with an enhanced risk of COVID-19 infection.

As previously mentioned, an urgent epidemiological effort has been undertaken to understand what risk factors are responsible for the principal outcome associated with COVID-19, i.e., mortality. From Chinese studies, mortality rates were increasing along age categories (mortality rate of 1.3% in the 50–59 range, 3.6% in the 60–69 range, 8% in the 70–79 range and 14.8% in the ≥range 80 years), the presence of cardiovascular diseases (10.5%), diabetes (7.3%), chronic respiratory diseases (6.3%), arterial hypertension (6%) and neoplasms (mortality 5.6%) [22,23]. In Italy, data from ISS—i.e., the Italian Health Institute—indicates that 1% of the patients who died did not suffer from any other diseases, 26% had only one disease, 26% had 2 diseases and 47% had 3 or more pathologic conditions. The most common chronic preexisting diseases in deceased patients were: arterial hypertension (70%), followed by diabetes mellitus (31.7%), chronic kidney disease CKD (23.1%), atrial fibrillation (22.5%), chronic obstructive pulmonary disease (COPD) (18.1%), the presence of an active cancer within the previous 5 years (16.8%), ischemic heart disease (16%) and obesity 10% [20]. It is very remarkable that CKD was present in more than 20% of the deceased patients due to COVID-19, also surpassing, in prevalence, those affected by COPD and/or those with an active cancer within the last 5 years.

In addition to the presence of CKD, it also has been demonstrated that COVID-19 patients are at increased risk of developing acute kidney injury (AKI), a clinical condition that is associated with unfavorable outcomes, including mortality [8,24]. The incidence of AKI in COVID-19 patients is similar to that found in SARS patients. This sudden loss of kidney function is strongly associated with increased mortality and morbidity and is a complication that can occur during the progression of COVID-19 in patients suffering from kidney disease as well as in those who are not [8,23,25]. From the analysis of several studies, this incidence varies from 0.5% to 23%, with an interval from baseline visit to the onset of AKI of 7–15 days in median [25,26]. Patients who developed AKI had a more critical prognosis, in terms of mortality rate compared with those who only had chronic illness as comorbidity (AKI increased the risk of death by 5.3 times in these patients) [27]. All these observations suggest that AKI could be one of the risk factors for mortality in COVID-19 patients, and a recent meta-analysis of 26 studies suggested that mortality from AKI may, in fact, be 13 times higher [28]. Overall, either the presence of CKD at hospital admission or the development of AKI during the COVID-19 infection have been both recognized as two independent risk factors of mortality [8,29,30].

COVID-19 has also affected patients on renal replacement treatments (RRT). The Italian Society of Nephrology (S.I.N.) report found 521 patients (2.8%) positive for SARS-CoV-2 in a population of 17,848 patients undergoing hemodialysis, 18 patients (0.65%) were positive in a population of 2252 patients treated with peritoneal dialysis. In positive RRT patients, 54% were dialyzed at their own dialysis center, 18% required hospitalization in sub-intensive care unit and 4.7% were dialyzed in an intensive care unit. Death occurred in a non-trivial proportion of cases (25.8%) [31]. Indeed, data from the Dialysis Units of Piedmont and Aosta Valley (two Italian Northern West regions) found that absolute mortality risk was higher in males than in females (31.1% vs. 4%, respectively) and in presence of cardiovascular disease (29.9% vs. 10.7%) whereas it was not related to dialysis vintage. Diabetes was a risk factor in transplanted patients (66.7% vs. 13.6%). A history of neoplasia was also associated with an increase in risk of death (40% vs. 19%) [32].

These data demonstrate how hemodialysis patients are particularly susceptible to COVID-19 infection.

## 3. Etiology

Coronaviruses are a group of single-stranded RNA viruses (ssRNAs) with positive polarity, belonging to the Coronaviridae family [33]. Until 2019, six coronavirus strains, which were able to infect humans, were known. Four strains usually circulate in the human population causing mild respiratory infections [34]. In 2003 and 2012, the first two zoonotic strains of coronaviruses capable of infecting humans through an animal were identified. These have caused severe lung syndromes in recent times: SARS in 2003 and MERS in 2012 [34]. Studies carried out on these strains showed that zoonotic coronaviruses use the bat as the primary host and are transmitted to humans through an intermediate host, represented by the civet for SARS and the camel for MERS [35]. However, some bat coronaviruses have been recently identified as being able to infect human cells without the need for an intermediate host [35].

The new respiratory infection originating from Wuhan (Hubei Province) in China that spread rapidly to the rest of the country, was initially traced back to Huanan Seafood Wholesale Market [13]; but only in January 2020 was a strain of coronavirus, which was unknown, identified as the responsible pathogen of the infection. It was named as SARS-CoV-2 and belongs to the β-coronavirus cluster that includes SARS-CoV and MERS-CoV [2]. SARS-CoV-2 is the third strain of zoonotic coronaviruses currently known [1]. Subsequently, it was documented that SARS-CoV-2 is a chimaeric virus resulting from pre-existing viruses: a bat coronavirus and another coronavirus of unknown origin [36]. Its genomic sequence corresponds to the bat coronavirus with an 88% identity [37] and the pangolin coronavirus with a 99% identity [38], although the genetic analysis performed on the pangolin coronavirus did not involve the whole viral genome, but a specific site known as the receptor binding domain [37]. However, it emerged that SARS-CoV-2 and the pangolin coronavirus did not share the same structural characteristics [39]. Therefore, pangolin was identified as the intermediate species of transition from bat to humans rather than directly responsible for the SARS-CoV-2 pandemic [3,11].

## 4. Pathophysiology

To understand the pathophysiological mechanism of SARS-CoV-2, its genomic sequence was compared to the other two similar coronaviruses, SARS-CoV and MERS-CoV. In fact, it emerged that SARS-CoV-2 has a sequence identity of 79% with SARS-CoV and 50% with MERS-CoV [37,40]. Upon analysis of certain proteins, such as the coronavirus main proteinase (3CLpro), papain-like protease (PLpro) and RNA-dependent RNA polymerase (RdRp), it was observed that the sequence identity value between SARS-CoV and SARS-CoV-2 is 96% [41]. Therefore, an analogy between the physiopathological mechanisms of SARS-CoV and SARS-CoV-2 has been hypothesized [42].

Many studies reported that SARS-CoV-2, like SARS-CoV, uses Angiotensin converting enzyme 2 (ACE-2) to enter target cells [43,44,45]. ACE-2 is a carboxypeptidase expressed on the cell surface which cleaves Angiotensin I (Ang I) into Angiotensin 1–9 and Angiotensin II (Ang II) into Angiotensin 1–7, counteracting the vasoconstrictor, proliferative and fibrotic effects of Angiotensin II generated by Angiotensin converting enzyme (ACE) [46]. Single-cell RNA sequencing analysis demonstrated a wide distribution of ACE-2 in different tissues [12,47] and histochemical staining then confirmed these data [48]. However, since low levels of ACE-2 expression were found on several cells types [47], it was supposed that cellular interaction and internalization by SARS-CoV-2did not depend only on ACE-2, but depended also on other auxiliary cell membrane receptors and proteins. In fact, it is recognized that ssRNA viruses tend to have multiple receptors [49]. Qi et al. analyzed the expression of ACE-2 on 119 cell types from 13 human tissues and the coexpression characteristics of the ssRNA human viral receptors and membrane proteins. Pearson correlation analysis of gene expression matrices showed 94 genes were found to be significantly correlated with ACE-2. Among these, the coding genes of the peptidases alanylaminopeptidase (ANPEP), glutamyl aminopeptidase (ENPEP) and dipeptidyl peptidase 4 (DPP 4) showed the highest correlation with ACE-2 [12]. While both ANPEP and DPP4 are already known as a target receptors for other coronaviruses (human coronavirus 229E, swine epidemic diarrhea virus, canine coronavirus, feline coronavirus, for ANPEP and MERS-CoV for DPP4), the relationship between ENPEP and viral infection is not yet known [12]. ENPEP is a member of the M1 family of endopeptidases which are mammalian type II integral membrane zinc-containing endopeptidases. It is mainly expressed in the terminal ileum and in the renal cortex and plays a role in the catabolic pathway of the Renin-Angiotensin system (RAAS), in the regulation of blood pressure and in the formation of blood vessels [50]. While the observations of Qi et al. suggest ENPEP may be a coronavirus receptor, further investigation is needed to confirm this [12].

However, the low expression of ACE-2 on the cell surface could also be interpreted as a viral defense mechanism. In the past, the down regulation of ACE-2 had been correlated with faster cell-cell spread of human coronaviruses [51] and with more severe clinical manifestations [52,53]. Guzzi et al., in a recent study, hypothesized that even for COVID-19 the down regulation of ACE-2 could be a mechanism induced by SARS-CoV-2 to obtain a faster intercellular diffusion [54]. Studies carried out to understand the effect of angiotensin receptor blocker (ARB) drugs in patients with COVID-19, have suggested mechanisms through which the upregulation of ACE-2 may be protective during SARS-CoV-2 infection [55]. ARBs, in fact, greatly increase the cellular expression of ACE-2 [56]. However, since SARS-CoV-2-ACE-2 interaction represents the first step of a chain of events, if the upregulation of ACE-2 is not followed by the increase of certain cell proteases essential for internalization and viral activation, it would only result in the sequestration of SARS-CoV-2 on the cell membrane limiting viral infection [35]. Furthermore, the metalloproteinase ADAM17 can act upon the membrane-bound ACE-2, leading to the release of a soluble form of ACE-2. If the increased expression of ACE-2 correlates with an increase in soluble ACE-2, this might act as a decoy receptor for SARS-CoV-2 by limiting viral entry into target cells [35].

Upon analysis of the SARS-CoV-2-ACE-2 interaction, there was confirmation that this occurs through the spike glycoprotein expressed on the viral envelope, being the same for all coronaviruses [35]. The coronavirus spike protein is composed of an intracellular segment, a transmembrane segment and a large ectodomain formed by an S1 subunit for interaction with the target receptor and an S2 subunit for fusion between the viral and cell membrane [57]. Subunit S1 consists of four domains, one N terminal domain (NTD) and three C-terminal domains (CTD1, CTD2 and CTD3). The cell receptor and the viral protein bind through the receptor-binding domain (RBD), located in the CTD1 domain in the case of SARS-CoV [58]. Experiments undertaken to investigate virus-receptor interaction with resolution at the atomic level showed that SARS-CoV and SARS-CoV-2 had a high sequence similarity (89.2%) and sequence identity (73.7%) [14,22]. However, a more targeted evaluation of SARS-CoV-2 RBD revealed peculiar characteristics that are probably responsible for the greater diffusion compared with SARS-CoV. Wan et al. found that the SARS-CoV-2 RBD has a single mutation that improves its binding affinity with ACE-2 [59]. Heet al. have shown that the characteristics of SARS-CoV-2 RBD make the virus more soluble, therefore capable of binding ACE-2 more easily, but also more stable, therefore able to survive at high temperatures [42]. At the same time, the SARS-CoV-2 RBD has greater flexibility than the SARS-CoV RBD, especially near the binding site. For this reason, SARS-CoV-2 is much more sensitive to temperature than SARS-CoV in terms of the RBD-ACE-2 bond and this would cause the decrease in infectivity with increasing temperatures [42]. The SARS-CoV RBD-ACE-2 binding induces conformational changes in the S2 subunit, such as to induce the fusion between the viral membrane and the cell. A low pH and pH-dependent endosomal cysteine proteases called cathepsins facilitate endosomal cell entry of the virus. Furthermore, the S protein is cleaved into the S1 and S2 subunits by the host transmembrane cell proteases [60], which are necessary for the entry of the virus through the cell surface non-endosomal pathway [61]. Hoffmann et al. have shown that in particular SARS-CoV-2 S protein depends on the cellular protease Transmembrane Serine Protease 2 (TMPRSS2) for priming [62].

Therefore, the coexpression of ACE-2 and TMPRSS2 is a determining factor for the entry of SARS-CoV-2 into the host cells [63]. After entering the cell and becoming activated, SARS-CoV-2 uses the endogenous transcription mechanism of the cells to replicate and spread [60]. Cells infected by SARS-CoV-2 can recruit and modulate immune cells through the secretion of chemokines or other cytokines [12]. The role of macrophages remains to be defined. In fact, the interaction between macrophages and cells expressing ACE-2 is known, suggesting a primary role of macrophages as a sentinel during viral infection [12]. A recent study, however, has shown a down regulation of mitochondrial proteins that interact with SARS-CoV-2. This mechanism could be interpreted as a process through which the virus prevents apoptosis induced by mitochondria [54].

## 5. Pathophysiology of Kidney Damage Induced by SARS-CoV-2

The expression of ACE-2 has been shown not only in the lung but also in the liver, stomach, ileum, colon, esophagus and kidney [47]. These data associated with the evidence that AKI (7%), myocardial dysfunction with acute cardiovascular events (12%) and gastrointestinal disorders are among the most frequent clinical manifestations of COVID-19 [23], suggesting that SARS-CoV-2 can infect these organs. However, whether SARS-CoV-2 replication occurs in these organs causing functional damage and contributing to the systemic spread of the virus is not yet clear.

Zou et al. [47], stratifying the human organs in high and low risk according to the level of ACE-2 expression, have shown that the kidney is very vulnerable to SARS-CoV-2infection.

Qi et al. [12], through single cell RNA sequencing studies, have shown that ACE-2 in the kidney is expressed primarily by renal proximal tubular cells (~82%) and also to a lesser extent by cells of the intercalated duct, main cells of the collecting duct, renal distal tubular cells, glomerular parietal epithelium cells and immune cells (8%) [12].

Pan et al. [63], using single cell transcriptome analysis, confirmed the cellular co-expression of ACE-2and TMPRRS genes in proximal tubule cells and podocytes.

Batlle et al. have also suggested whether the expression of other cellular TMPRSSs other than TMPRSS2 (such as TMPRSS 4, 5 or 9) may also play a role in the priming step [64]. Studies showed that the co-expression of the ACE-2 receptor and TMPRSS genes in kidney epithelial cells was as significant as in the lung, esophagus, small intestine and colon, suggesting that the kidney might also be an important target organ for SARS-CoV-2 [63]. In particular, a high co-expression of ACE-2 and TMPRSS genes was found in podocytes and proximal rectangular tubular cells [63].

Other noteworthy observations include the presence ofSARS-CoV-2 nucleocapsid protein in renal tubular structures and virus-like particles in podocytes and renal tubular epithelial cells, as observed by electron microscopy [65,66]. Together these observations suggest the virus may cause AKI through a direct cytopathic effect on kidney cells. In particular, it is conceivable that the virus may enter the kidney by invading the podocytes first, thereby gaining access to the tubular fluid and thence to the proximal tubule cells where it may bind to ACE-2 [64]. The viral replication in podocytes and the resulting damage could explain the proteinuria and hematuria reported in a high percentage of COVID-19 patients [8,27]. However, if the renal dysfunction is caused only by direct damage of SARS-CoV-2 or is secondary also to other systemic processes triggered by the virus, it has not been well defined yet.

Diao et al. [65] observed that kidney damage associated with COVID-19 is an acute tubular necrosis induced directly by SARS-CoV-2 during infection and replication, but also indirectly through the complex immune mechanisms triggered by cellular damage. In fact, the histopathological examination performed on kidney specimens, obtained from autopsy of COVID-19 patients with renal function impairment, showed viral antigens in the cytoplasm of the tubular cells, but also a strong presence of CD68^+^ macrophages in the tubulo-interstitium and strong C5b-9 depositions on the apical brush border of tubular epithelial cells (TECs). This suggested that proinflammatory cytokines derived from macrophages in the tubulo-interstitium and complement-mediated mechanisms resulting from cell damage participate in the pathogenesis of tubulo-interstitial damage. In fact, despite the infiltration of infected tissue by host immune cells in order to contain viral replication, the hyperactivation of these immune cells may lead to fibrosis, epithelial cell apoptosis and cause microvasculature damage [67,68,69].

Studies performed on SARS-CoV suggested that AKI in SARS patients was the result of specific pathogenic conditions, such as the cytokine release syndrome [70], rather than active viral replication in the kidney. In consideration of the analogy between SARS-CoV and SARS-CoV-2, flow cytometry was used to study the immune phenotype and the function of peripheral blood mononuclear cells in COVID-19 patients [71]. Studies showed that patients infected by SARS-CoV-2 showed lymphopenia, mainly related to the significant reduction in absolute T cell counts, particularly cytotoxic T lymphocytes (CD8^+^), increased neutrophil counts and elevated levels of proinflammatory cytokines. In particular, high levels of interleukin (IL)-2, IL-6, IL-10 and interferon (IFN)-γ were observed. Therefore, it has been speculated that a loss of T cells during the viral infection may result in enhanced inflammatory responses [72]. In fact, it is known that T cells are important for dampening overactive innate immune responses during viral infection [73]. In accordance with this hypothesis, it has been observed that when the T cell count drops the serum levels of IL-2, IL-4, IL-10, tumour necrosis factor (TNF)-α and interferon (IFN)-γ reach their peaks [72].

Another important finding is that COVID-19 patients with more severe clinical manifestations have higher serum concentrations of IL-6 and lower IFN-γ than mild forms. This is mainly due to the decrease in CD4^+^, CD8^+^ and NK lymphocytes [74]. Interferons (IFN) are a family of cytokines that play a central role in innate immunity to viruses and other microbial pathogens. The IFN-receptor binding induces a cascade of signals with activation of genes coding for proteins with antiviral, antiproliferative or immunomodulatory properties [70,75]. Normally the interaction between the IFN-γ and IL-6/sIL-6R signals contributes to the recruitment and subsequent clearance of neutrophils, thereby controlling infection and resolution of acute inflammation as well as influencing the transition between innate and adaptive immunity [76]. In patients infected by SARS-CoV-2, a higher IL-6/IFN-γ ratio may be related to an enhanced cytokine storm [74].

These observations suggest that in both SARS patients, and in patients with COVID-19, AKI may have an inflammatory etiology mediated by a cytokine storm. 

The cytokine storm is associated with an inflammatory process that originates in a local site and spreads via the systemic circulation. The inflammatory process can cause dysfunction in organs, particularly when tissue edema causes an increase in extravascular pressures and a consequent decrease in tissue perfusion. Compensatory repair processes arise soon after the beginning of inflammation, and in a lot of cases they can completely re-establish tissue and organ function. However, when a severe inflammation condition injures local tissue structures, healing occurs with fibrosis, which can cause permanent organ dysfunction [70]. In fact, when a cytokine storm occurs, the immune system may not be able to kill SARS-CoV-2, but it can kill large numbers of normal cells and damage organs [63]. In support of the hypothesis that AKI in COVID-19 patients may be the consequence of inflammatory damage, a cohort study found that the CT scan of kidneys showed a reduced density, indicative of inflammation and edema [8].

In addition to being frequently associated with the cytokine storm, severe lung infections often require prolonged ventilatory support. This predisposes to the development of sepsis, classically defined by marked hypotension which requires treatment with inotropic drugs. Therefore, in patients with COVID-19 or acute respiratory distress syndrome (ARDS), it is plausible that persistent hypotension and vasoconstriction induced by inotropics can participate in the fall of the glomerular filtrate and consequent acute tubular necrosis [77].

In the most recent studies, the hypothesis of a multifactorial etiology of renal damage in COVID-19 is confirmed. Su et al. [66] in a study performed by analyzing autopsy kidney samples showed pigmented casts with high levels of creatine phosphokinase, attributable to rhabdomyolysis. In rhabdomyolysis the massive release of myoglobin due to muscle damage can cause kidney dysfunction. Myoglobin, in fact, shows its renal toxicity through various mechanisms: renal vasoconstriction related to the hyperactivation of RASS by hypoperfusion and the reduction of nitric oxide levels; intratubular cast formation; direct toxicity on renal tubular cells. These processes result in acute tubular necrosis [78]. Rhabdomyolysis in COVID-19 patients is hypothetically multifactorial. In fact, it may be secondary to a direct cytotoxic effect of SARS-CoV-2 on the muscle, tissue hypoxia due to hyperventilation or also to drug-induced damage [66]. Presumably, in COVID-19 patients who develop rhabdomyolysis, it may participate in the pathogenesis of AKI.

Su et al. [66] demonstrated the presence of erythrocyte aggregates, without platelets or fibrinoid fragments, which obstructs the lumen of the peritubular and glomerular capillaries in COVID-19 patients. Erythrocyte aggregation, presumably induced by inflammation (reflected by a high rate of erythrocyte sedimentation) and hypotension, can potentiate oxidative stress, inflammation and complement activation, aggravating microvascular damage [64]. Furthermore, occlusion of microvascular lumens by erythrocytes has been associated with a variety of endothelial lesions [66]. Normally, in endothelial cells of the kidney, only ACE is expressed without detectable ACE-2 [79]. Therefore, the renal endothelium cannot be infected directly by SARS-CoV-2. However, this cannot be totally excluded, since ACE-2expressioncan be changed in pathological states or by drugs [66]. Varga et al. recently concluded that SARS-CoV-2 infection induces endothelitis in various organs, directly and indirectly, and that could explain the systemic impairment of microcirculation [80]. Further studies are necessary to better understand the genesis of renal endothelial lesions. Of note, a recent study has proposed a new route for SARS-CoV-2 invasion of host cells via an alternative cell receptor known as CD147 (and also called basigin), which is a transmembrane glycoprotein and is expressed on all endothelial cells [81]. 

Recently, the high incidence of thromboembolic events in COVID-19 patients suggests that SARS-CoV-2 may play an important role in inducing coagulopathy.

Analyzing the hematological profile of COVID-19 patients, a state of hypercoagulability emerged. In fact, high plasma levels of reactive protein C, fibrinogen, D-dimer and ferritin, associated with thrombocytopenia, were found in these patients [71,82]. Recently, clinical and autopsy reports from China and the U.S. confirm the development of disseminated intravascular coagulation following SARS-CoV-2 infection, with evidence of microangiopathy in several organs. In fact, the activation of macrophages associated with COVID-19, the storm of cytokines and the molecular proteins associated with the damage can cause both tissue factors’ release and the activation of coagulation factors predisposing to hypercoagulability.

In some cases, this state of hypercoagulability could favor the evolution of acute tubular necrosis into cortical necrosis and, therefore, the development of irreversible renal damage. These observations suggest that low back pain and microhematuria observed in some positive COVID-19 patients may be manifestations of renal infarction [64].

The SARS-CoV-2 contribution to the development of CKD could involve pathways similar to those described for the acute kidney injury. Indeed, it has been observed that a non-trivial portion of patients develop signs of tubular or glomerular damage during the infection. The direct tubule-glomerular cellular injury, due to the virus, often manifests with proteinuria and hematuria that, in turn, could start a chronic, non-reversible, process [83]. It has been shown that proteinuria exerts a direct toxic effect on renal tubular cells and promotes renal fibrosis over time [84,85].

**Figure 1:**
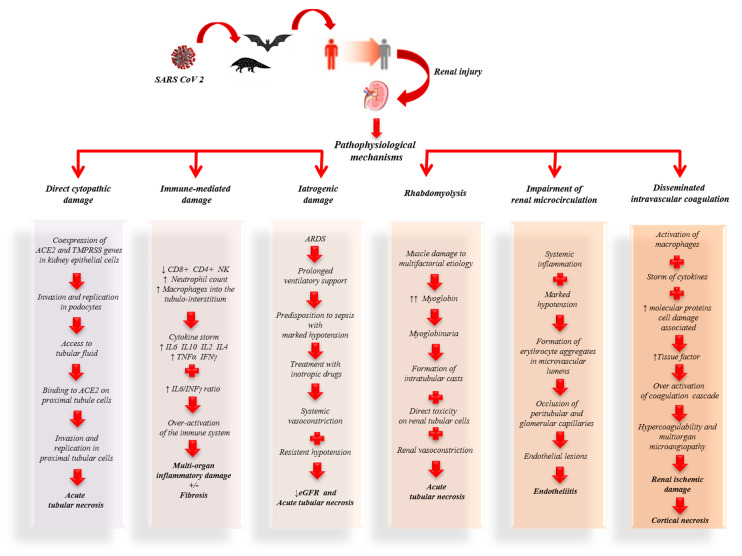
Pathophysiological mechanisms of kidney damage associated with coronavirus disease 2019 (COVID-19).

In conclusion, the renal damage observed in COVID-19 patients is the result of complex mechanisms induced directly and indirectly by SARS-CoV-2 that predispose to the development of renal dysfunction (Figure 1).

Further studies are needed to better understand the pathophysiological mechanisms of kidney injury, to develop new therapeutic strategies able to limit and/or prevent kidney damage, and to improve the prognosis of COVID-19 patients.

## 6. Histopathology

As mentioned before, the virus gains access to the kidney via the ACE-2receptor for which it uses to enter entrance to target cells [62,86,87] and it has been shown that the ACE-2 receptor of SARS-CoV-2 is highly expressed in renal proximal tubule cells [63,88,89]. Possible mechanisms for kidney injury in COVID-19 include direct infection of the renal parenchyma [47].

Diao et al. [65] retrospectively analyzed the clinical data on renal function from 85 cases of COVID-19; a similar analysis on kidney abnormalities in 26 autopsies of COVID-19 patients was conducted by Su et al. [66]. Using light microscopy, ultrastructural observation and immunostaining have been very informative to understand the extent of kidney injury.

Light microscopy examination using Hematoxylin and Eosin staining may show the extent of tubular atrophy and interstitial disease, these are strong histological markers of renal damage and may also predict progression of renal failure. Using light microscopy, proximal tubule injurywas observed with the loss of brush border and vacuolar degeneration with necrosis and detachment of the epithelium observed in the lumen of the tubules; dilatation of the tubular lumen was also noted together with cellular debris resulting from necrosed cells. Erythrocyte aggregates were also observed, causing obstruction of peritubular and glomerular capillary loops, with no obvious erythrocyte or platelet fragmentation or any fibrin thrombi, and less erythrocyte aggregation was observed in peritubular capillaries in those cases with predominantly glomerular loop obstruction [65,66].

Hemosiderin granules were identified in the renal tubular epithelium of a few patients, while pigmented casts were found in a small number of patients who had high levels of creatine phosphokinase, indicating the presence of rhabdomyolysis. Su et al. noted some cellular swelling of the renal distal tubules and collecting ducts, with some edematous expansion of the interstitial space, but without any significant inflammation. They also noted infiltration of lymphocytesin areas of nonspecific fibrosis, including subcapsular areas. Glomeruli were intact, with various degrees of morphologic changes: nodular mesangial expansion and arteriolar hyalinosis; endothelial cell swelling and degeneration was observed in a small number of COVID-19 patients but these were older individuals with a history of diabetes and hypertension; in other cases podocyte vacuolation and detachment from the glomerular basement membrane was seen; in 2 patients with proteinuria and diabetes, focal segmental glomerulosclerosis was also noted. 

Immunohis to chemical studies utilizing an anti-SARS-CoV nucleoprotein antibody against the viral nucleocapsid protein (NP) and indirect fluorescence microscopy, showed the NP antigen was present in the renal tubular cells of the infected tissues, suggesting that SARS-CoV-2 can directly infect kidney tubules. Moreover, Diao et al. observed that the presence ofSARS-CoV-2 virus resulted in the infiltration of high levels of CD68^+^ macrophages into the tubulo-interstitium, and it is feasible that these macrophages could release proinflammatory cytokines which in turn would cause renal tubular damage. In contrast, CD8^+^ T cells were observed only in moderate numbers in the examined tissues, while CD4^+^ T cells and CD56^+^ natural killer (NK) cells were seldom found.

Diao et al. also observed the deposition of the C5b-9 complement complex (also known as the membrane attack complex pathway) on the apical brush border of renal tubular epithelial cells, whereas C5b-9 expression was absent in normal kidney tissue. The complex may cause cell death and therefore renal injury and may be an important factor in the pathogenesis of tubulo-interstitial damage [90,91]. All these findings suggest that SARS-CoV-2 infection causes acute tubular necrosis not only through direct cytotoxicity, but also via immune mediated mechanisms.

Observations using transmission electron microscopy (EM) of kidney tissue from autopsies of COVID-19 cases has shown the presence of coronavirus-like particles in the cytoplasm of proximal and distal tubular cells; as well as in podocytes, podocyte foot processes and the glomerular basement membrane. In two autopsy specimens observed by Diao et al., it was noted that the cells in infected kidney tissue were swollen, with enlargement of mitochondria and lysosomes, and viral particles were observed in the broken lysosomes in the cytoplasm. These findings demonstrate SARS-CoV-2 can directly infect kidneys and why patients with COVID-19 often present with proteinuria, hematuria and AKI.

**Figure 2:**
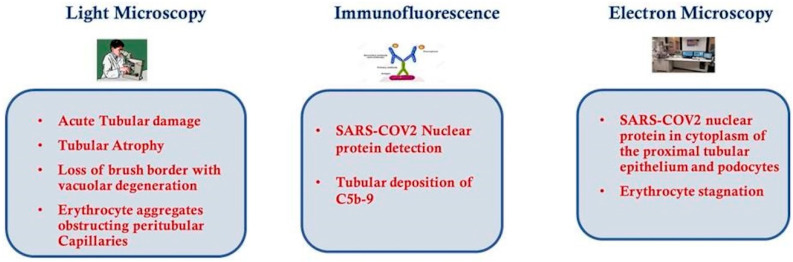
Histological features of kidney from COVID-19 patients.

As has already been mentioned with the light microscopy observations, EM also showed erythrocytic obstruction of peritubular capillary lumens with injury of the endothelium. Aggregation of erythrocytes in segmental glomerular capillary loops was frequent, but without inflammation or necrosis. Intriguingly, according to a recent study [14] about endothelial cell infection and endothelitis in COVID-19, endothelial dysfunction is probably one of the main causes of microvascular dysfunction by causing vasoconstriction leading to organ ischemia, inflammation with associated tissue edema and a procoagulant state. The principal findings of light microscopy, immunofluorescence and EM are shown in Figure 2.

## 7. Clinical Features

Most COVID-19 infections are not severe, with the spectrum of symptoms ranging from mild to critical.

Based on all confirmed, suspected and asymptomatic cases of COVID-19 in the world on 23 July, the ISS—i.e., Italian Health Institute, reported that: 80% of infections are mild(asymptomatic 29% or with flu-like symptoms paucisymptomatic 12%, mild 35%), and those with these symptoms being able to recover at home; 10% are severe, developing severe diseases including pneumonia and dispnea; and 2% are critical and include: respiratory failure, septic shock and multi-organ failure requiring intensive care assistance; in about 2% of overall reported cases, the virus is fatal [92]. Comorbidities which have been associated with illness severity and mortality include the following ones: diabetes mellitus, cardiovascular disease, hypertension, chronic lung disease, cancer, chronic kidney disease, immunocompromising conditions, severe obesity (body mass index ≥ 40) and liver disease [90,91]. COVID-19 infection is frequently severe among patients of advanced age and other medical comorbidities. Males, compared with females, suffer a disproportionately higher number of deaths according to data from cohorts of patients in China, Italy and the United States [15,92].

The most frequent serious clinical manifestation of infection appears to be pneumonia, which is primarily characterized by fever (which can even be absent), dry cough, fatigue, anorexia, myalgias, dyspnea, sputum production, and the presence of bilateral infiltrates on chest imaging. Other reported symptoms can include the following: headache, sore throat, rhinorrhea and conjunctivitis [93]. However, no specific clinical features that can reliably distinguish COVID-19 from other viral respiratory infections have been reported, although development of dyspnea several days after the onset of initial symptoms is suggestive [25]. However, common symptoms in patients infected by COVID-19 are smell and taste disorders (e.g., anosmia and dysgeusia) [93]. Gastrointestinal symptoms (e.g., nausea, vomiting, abdominal pain and diarrhea) have also been described [94]. There have been rare dermatological reports of erythematous rash, widespread urticaria and chickenpox-like vesicles and transient livedo reticularis [11]. Reddish-purple nodules on the distal digits similar in appearance to pernio (chilblains) have also been anecdotally described in children and young adults with suspected COVID-19 infection. 

As mentioned previously, epidemiological data from many countries report that children make up a small minority of those who test positive. Children account for 1–5% of patients and are less likely to become severely ill compared with adults, though preschool children and infants might have severe clinical features [95,96]. The small rate of COVID-19 infection for children has also been confirmed by another study which showed that children younger than 18 years made up less than 2% of national cases in different countries [97], so that, also in this case, that proportion reflects lower susceptibility among children versus adults [98]. The emergence of a severe Kawasaki-like disease in children related to COVID-19 has now shifted focus on the vulnerability of children [99]. It is a rare acute pediatric vasculitis, with the development of coronary artery aneurysms as its main complication. Diagnosis of this disease is based on the presence of persistent fever, lymphadenopathy, conjunctival injection exanthema and changes to the mucosae and extremities. Pediatricians in the United Kingdom identified a small group of children presenting with shock and a multisystem inflammation, some of whom had coronary artery aneurysms, and a further group of less severely ill children with a Kawasaki-like disease. Based on the review of clinical and laboratory features, a case definition of the syndrome named “pediatric inflammatory multisystem syndrome temporally associated with SARS-CoV-2 (PIMS-TS)” was formulated by experts in the United Kingdom and published by the Royal College of Paediatrics and Child Health [100].

Some COVID-19 patients who at first do not show anysevere symptoms may nonetheless do so over the course of a week, with acute respiratory distress syndrome (ARDS) manifesting shortly after the onset of dyspnea in patients with severe disease. Other reported complications are acute cardiac injury, arrhythmias, AKI and shock [25]. Thromboembolic complications, including pulmonary embolism and acute stroke, have also been reported [101].

### 7.1. Clinical Features in Patients with Chronic Kidney Disease (CKD) 

Chronic kidney disease (CKD) seems to be associated with enhanced risk of severe COVID-19 infection and mortality. Cheng et al. evaluated the association between markers of renal impairment and death in a cohort of 701 COVID-19 patients. They found that 43.9% of patients admitted had proteinuria and 26.7% had hematuria, serum creatinine and blood urea nitrogen (BUN) levels were increased in 14.4% and 13.1% of patients, respectively. Estimated glomerular filtration rate < 60 mL/min per 1.73 m^2^was found in 13.1% of patients [8]. In particular, the authors have shown that, at univariate analysis the presence of proteinuria was associated with a 4 up to 11-fold increased risk of in-hospital death compared with COVID-19 patients without kidney damage, whereas hematuria increased the risk of death by 12 times. These hazard ratios (HR)were higher than risk factors such as advanced age (HR: 2.43), severe disease (HR: 6.10) and remained significantly associated with mortality even after adjustment (therefore to multivariate analysis) by age, gender, disease severity, lymphocyte count, comorbidity, thus demonstrating that measures of kidney damage play a very important role in assessing prognosis of COVID-19 patients [8,102,103]. 

This significant association of CKD with severe COVID-19 infection was observed also in the meta-analysis by Lippi [21]. This can be explained by the pro-inflammatory state and by the alterations of the innate and adaptive immune response associated with CKD. This immune profile increases susceptibility to all infections [104]. These findings suggest that COVID-19 patients with high baseline serum levels of creatinine are more likely to be led to intensive care unit treatment and to undergo mechanical ventilation, because the presence of a renal disease on admission constitutes a higher risk of negative prognosis. It has been recently shown that a large part of COVID-19 patients suffer from other comorbidities and most of these patients are also elderly and males [102,103]. Among these comorbidities, the presence of chronic kidney disease is an independent risk factor of poor prognosis. It is also true, on the contrary, that nephropathic patients are mainly affected by hypertension and cardiovascular disease per se and this can lead to a higher risk of COVID-19 infection when compared with the general population or with patients without kidney disease [105]. Nephropathic patients are also patients with cardiovascular disease which is currently considered a biomarker of increased risk for COVID 19 infection and for poor prognosis [105]. However, an increased risk of death, about 3–8 times, was found in patients infected with other viruses such as H1N1 flu virus and who developed kidney injury during infection as compared to those who had not [106]. Moreover, patients who enter the hospital with elevated serum creatinine levels were predominantly male and older (median age was 73 years) and were more severely ill compared with patients who had normal serum creatinine (median age was 61 years). In addition, patients with increased baseline serum creatinine levels show an alteration of leukocyte count with an increase in the absolute number of leukocytes and a decrease in lymphocyte and platelet counts. Coagulation pathway abnormalities, which include prolonged activated partial thromboplastin time and higher D-dimer, are more frequent in patients with increased baseline serum levels of creatinine. The rate of patients with increased procalcitonin, and the plasma levels of aspartate aminotransferase and LDH are also higher in patients with CKD compared with those with normal renal function. The incidence of in-hospital death in patients with CKD was found to be significantly higher than in those patients with normal baseline serum levels of creatinine.

### 7.2. Clinical Features in Patients with Acute Kidney Injury (AKI)

It is not the first time that a virus mainly involving the respiratory tract can also involve the kidney, as it has been already reported during the course of the SARS epidemic in 2003 [8,107]. AKI represents a life-threatening complication, often leading to increased risk of death. One possible explanation of the high prevalence of kidney involvement at hospital admission is that some of the COVID-19 patients may already have had a history of CKD. Such patients tend to have a pro-inflammatory state with functional defects in their immune system [108] and are at a higher risk for upper respiratory tract infection and pneumonia. Cheng et al. [8], as mentioned before, in their study of a cohort of 701 patients found that 5.1% of patients developed AKI during hospitalization. Patients with increased baseline serum creatinine levels were more likely to develop AKI (11.9%) than patients with normal baseline values (4.0%). This means that, while renal complications are more likely in patients with pre-existing chronic impairment of kidney function, moderate-to-severe AKI can also be found in patients with normal serum creatinine levels these may represent a higher-risk subset of patients with ARDS. Wilson et al. [109] noted that similar observations have been reported for COVID-19-associated ARDS, which could develop into AKI on average 9 days after admission together with secondary infections and acute cardiac damage [26]. In ARDS, patients age, severity of illness and the presence of diabetes are all risk factors for acute kidney injury. Furthermore, the patient’s BMI value and any previous history of heart failure may also be associated with the severity of AKI. All these risk factors may count for the higher incidence of AKI in the elderly. Hirsch et al. recently analyzed risk factors, clinical presentation and outcomes of AKI among hospitalized COVID-19 patients in the metropolitan New York area, encompassing twenty three hospitals within urban and suburban areas and including academic tertiary and community hospitals. They found that among those with AKI, 694 died (35%), 519 (26%) were discharged and 780 (39%) were still hospitalized. In addition to the known risk factors for AKI mentioned before, namely older age, cardiovascular disease, hypertension, diabetes mellitus and need for ventilation and of vasopressor drugs, black race was also included among them. Indeed, individuals from minority communities, in particular African Americans and Hispanics have been disproportionately affected by and have had worse outcomes after SARS-CoV-2 infection. Finally, they did not find that use of blockers of the Renin-Angiotensin and aldosterone system at hospital admission for COVID-19 disease was associated with greater AKI risk. As expected, in their study involving different ethnicities in U.S.A., they confirmed that early AKI occurs frequently among COVID-19 patients and in temporal association with respiratory failure, with a consequent poor prognosis [110].

The standard assessment of AKI is still based on serum creatinine levels and daily urine output, but these represent only indicators of established renal damage [111]. Recently, Richardson et al. enrolled in the New York City Area (U.S.A.), the largest number of cases of sequentially hospitalized patients with confirmed COVID-19 in USA. Males and those with pre-existing hypertension and/or diabetes were highly prevalent among 5700 case series with a median age of 63 years. In this study, AKI was observed among 8.4% discharged live patients and among 63% dead patients. On hospital admission, a significant percentage of patients had renal impairment, presenting proteinuria and hematuria. AKI incidence in the overall cohort was in the range of 4.7–7.5%. A higher incidence of proteinuria and hematuria was reported in patients with severe or critically ill COVID-19 pneumonia. Among all patients with renal impairment, the patients with AKI had a higher incidence rate of proteinuria and hematuria compared with the non-AKI group. Almost 50% of the critically ill cases developed AKI during hospitalization, especially those who were in the intensive care unit (ICU). Patients were followed up for a median duration of 12 days, during which time most of the COVID-19 patients showed remission of the pneumonia. Urine dipstick testing in most of the patients with proteinuria and hematuria were reported as negative after follow-up. The mean time for AKI recovery was seen to be 6 days. The percentage of patients who developed AKI was increased in patients with diabetes. In conclusion, despite the high morbidity of kidney impairment, the short-term renal prognosis of those patients is still good: in fact 50% achieved remission in 3 weeks after the onset of their symptoms. However, adverse short-term outcomes of patients with kidney impairment are also associated with high rate of mortality in COVID-19 infected patients [15].

The pattern described by Richardson regarding AKI was similar to data reported from China: a study [112] of 138 patients with COVID-19 reported that ~4% of COVID-19 patients had AKI. Huang et al. [23] reported that among 41 patients with COVID-19 infection, 10% had elevated serum creatinine levels on admission and 7% had suffered AKI. In addition, both blood urea and serum creatinine levels progressively increased along the course of COVID-19 infection. In comparison with patients with normal serum creatinine levels, those who entered the hospital with high serum creatinine levels were predominantly male and old and were more severely ill too. Another study [27] involving 193 patients with COVID-19 infection has reported that, at hospital admission, 59% of the patients had proteinuria, 44% hematuria, 14% increase in BUN plasma levels and 10% increase in serum creatinine values. The abdomen CT showed kidneys with reduced density, suggestive of inflammation and edema of the renal parenchyma. Li et al. also observed that as many as 28% of COVID-19 patients developed AKI (9% of non-severe patients and 66% of severe patients). During hospitalization with a median of 2 days, there was an increase in BUN plasma levels in 30% of patients and, with a median of about 5 days, there was an increase in serum creatinine levels in 22% of patients. As already mentioned, COVID-19 patients with AKI have a higher mortality risk than those without AKI [27,28]. In contrast, patients with chronic illnesses (cardiovascular and cerebrovascular diseases, nervous system diseases, respiratory system diseases, digestive system diseases, urinary system diseases, reproductive system diseases and endocrine system diseases) had only on average ~1.5 times mortality risk. Furthermore, one study reported a 100% mortality rate for patients with stage 3 AKI [113]. Therefore, the presence of a renal impairment in COVID-19 patients is an important negative prognostic factor for their survival. However, dissimilar conclusions have been reported by Pei et al. who in a retrospective study, finding renal abnormalities in most of the patients with COVID-19 pneumonia, showed that renal complications in COVID-19 patients were associated with higher mortality, although proteinuria, hematuria and AKI often resolved in such patients within 3 weeks after the onset of symptoms [114]. If conservative treatments fail, RRT should be considered in patients with volume overload, especially those with refractory hypoxemia. In patients with COVID-19 and AKI, early initiation of RRT and sequential extracorporeal organ support (ECOS) seem to provide adequate organ support and seem to prevent the worsening of the disease’s severity [115]. A high degree of AKI has been reported to require extracorporeal therapies, such as (RRT) in critically ill patients, preferentially utilizing CRRT (continuous renal replacement therapies) for patients in the ICU [115]; recently Nalesso et al. have reported to have designed a continuous veno-venous hemodialysis (CVVHD) with a high cut-off membrane (HCO) in regional citrate anticoagulation (RCA) named by them as RCA-HCO-CVVHD. This treatment gives several advantages, when comparing it with an equivalent dose in continuous veno-venous hemofiltration, which include a lower effluent volume, fewer bag interventions (hence reducing nurses’ time for this), lower filtration fractions with a higher filter and circuit lifespan and fewer complications with blood flow as a result of central venous catheter malfunction [116]. Grasselli et al., in a study conducted in Italy and involving 1591 ICU patients with COVID-19, have reported that 27% of them required prone ventilation and that Extra Corporeal Membrane Oxygenation (ECMO) was performed in 1% of these patients [117].

### 7.3. Clinical Features in Patients in Hemodialysis (HD)

The ERA-EDTA has created a European database that collects individual data of dialysis patients with COVID-19; in the last update dating back to 23rd July, 5596 cases of COVID-19 and 1331 deaths were reported among hemodialysis patients [118].Patients undergoing dialysis have impaired immune systems and have a high risk of infectious disease. Kwan et al. [119] reported that dialysis patients had a higher rate of contracting SARS compared with an individual from the general population, but both the degree of disease severity and the mortality rate of the dialysis group were similar to the one of the control group. Based on SARS-CoV-2′s sequence similarity with SARS, it is possible that COVID-19 could follow the same trend as the one of SARS in patients undergoing hemodialysis. To understand the clinical features of COVID-19 infection, it is of interest to consider the first multicenter study focused on 7154 patients undergoing long term hemodialysis in 65 centers in Wuhan (China) [120]. Among all patients in HD, 154 had laboratory-confirmed COVID-19 tests with an incidence of COVID-19 infection in 2% of patients, which is much higher than that of the general population. This may be explained with the advanced age of the dialysis population, to the altered immune systems because of their uremic condition and the significant comorbidities that accompany this group, like cardiovascular disease, diabetes and cerebrovascular disease. Among 154 patients with COVID-19, 77% were mild/moderate patients, whilst 23% were severe/critical patients. The primary causes of end stage kidney disease (ESKD) in patients with severe/critical disease were similar to those with mild/moderate disease. The presence of cardiovascular disease was more likely to be present in patients with severe/critical infectious disease than among those with mild/moderate disease. Moreover, there were no significant differences between severe/critical and mild/moderate COVID- 19 infection regarding age, gender, smoking status and complications like diabetes, chronic obstructive pulmonary disease and cancer. The dialysis model, access, frequency of dialysis had no significant effect on the degree of COVID-19 disease severity. The most common symptoms, especially in the severe/critical group, were: fever, cough, fatigue, sputum production, dyspnea, nausea/vomiting, diarrhea and sore throat. An interesting case report of one of the first patients with COVID-19 in ESKD suffering from gastrointestinal (GI) problems highlights the importance of considering other clinical presentations of COVID-19 infection and not just focusing on the typical respiratory symptoms, in order to prevent exposure of potentially affected individuals to the general population. GI symptoms are somewhat unusual and seem to be a delayed clinical presentation of COVID-19 infection [121]. However, some of those symptoms may be difficult to distinguish from uremic symptoms. Diabetic patients showed more likely symptoms when infected. The disease symptoms of hemodialysis patients were similar to those of the general population, but only 50% of them had fever and nearly 25% were asymptomatic over the whole clinical course of COVID-19 infection. Since hemodialysis patients have disorders of B- and T-cell function [122], patients may show atypical clinical presentations. The most common finding on chest computed tomography was ground-grass or patchy opacity. These lesions often involved both the lungs; on the other hand, consolidation in lungs was not common. These radiological findings were similar to those shown in the general population, but sometimes it was difficult to distinguish them from lung changes due to their uremia status or inadequate dialysis.

Data from laboratory tests showed that lymphocytopenia was common in patients described in other reports [26,120], and there was a trend for the decline of lymphocytesin the severe/critical group, which is consistent with the results of other recent clinical reports. The uremic state of these patients is associated with a wide range of impairments in lymphocyte and granulocyte functions; lymphocytopenia is also common in dialysis patients. Considering low lymphocyte counts in chronic hemodialysis patients [108], lymphopenia is unlikely to be helpful for identifying individuals infected by SARS-CoV-2. Procalcitonin levels have similar limitations: in fact, its levels are chronically elevated in hemodialysis patients, even in the absence of severe acute illness. In addition, the majority of patientshad normal white cell and platelet counts. Serum albumin levels were also within the normal range for most of the patients too. Conversely, considering these limitations and the high prevalence of comorbid conditions, COVID-19 pneumonia diagnosis in hemodialysis patients is based on clinical epidemiology, radiographic findings and viral nucleic acid testing [123]. Interestingly, diabetes, as a primary cause of ESKD or a coexisting disorder, was much more common in symptomatic patients affected by COVID-19 and lymphocytopenia was also more severe. In total, about 40% of patients had at least one compromised organ, including cardiac injury, liver dysfunction, ARDS and a cerebrovascular event. Most patients undergoing hemodialysis (HD), who were regularly monitored, showed stable clinical conditions in the course of treatment of COVID-19 and pulmonary inflammation was gradually absorbed.

### 7.4. Clinical Features in Peritoneal Dialysis Patients (PD) 

The Italian report created by SIN has collected individual data of peritoneal dialysis (PD) patients with COVID-19; in the last update dating back to May, 57 cases of COVID-19 and 28 deaths were reported in total. This report shows a higher rate of mortality, almost 49%, than the general population [124]. Notably, SARS-CoV-2 positivity corresponded with peritoneal dialysis failure, suggesting a possible effect of the virus on the peritoneal membrane. The first case in the literature which demonstrated SARS-CoV-2 in the peritoneal fluid, was described during an emergency surgical laparotomy in a COVID-19 sick patient. During the procedure laparotomy, two swabs were obtained from peritoneal fluid and then sent for SARS-CoV-2 detection by specific real-time reverse transcriptase–polymerase chain reaction targeting three SARS-CoV-2 genes. It was then detected in the peritoneal fluid at a higher concentration than in the respiratory tract [125]. Recently, an Italian study described for the first time the detection of SARS-CoV-2 in the peritoneal waste of a patient with COVID-19 and ESKD on peritoneal dialysis. The patient showed the following common COVID-19 infection symptoms: fever, cough, myalgia, headache and mild hypoxemia. Chest computed tomography reported bilateral multiple ground-glass opacities and laboratory tests showed mild lymphopenia, increased C-reactive protein and D-dimer levels. The patient’s peritoneal dialysate was tested for SARS-CoV-2 by polymerase chain reaction, and was found to be positive [126]. According to the International Society Peritoneal Dialysis (ISPD) strategies regarding COVID-19 in peritoneal dialysis patients, the management of infection is the same for PD patients as for all other patients. Mild or moderate patients on PD can continue PD treatment as usual, with prescription adjustment according to the usual general evaluation. Those cases that aresevere or critically severe and requiring life support due to multiple organ dysfunction syndrome, can be temporarily transferred to automated peritoneal dialysis or bedside continuous kidney replacement therapy (CKRT). As for patients on hemodialysis, it is advisable to keep patients in a ‘dry’ status, so that an increased ultrafiltration may be required if remaining on PD [127].

### 7.5. Clinical Features in Kidney Transplant Patients

The European database created by ERA-EDTA [118] (collects individual data of dialysis patients and transplant patients with COVID-19; in the last update dating back to 23 July, 1403 cases of COVID-19 and 276 deaths were reported in total. Similarly, the last Italian report of SIN observed 218 kidney transplant patients affected by COVID-19, of whom 54 died [124]. Both reports show a higher rate of mortality (ERA-EDTA 19% and SIN 25%) than the general population. Moreover, as a population living with immunosuppression, the clinical manifestations, treatment and prognosis of COVID-19 pneumonia for renal transplant recipients may differ from those of the general population [128]. The immune response of renal transplant recipients, particularly the T-cell immune response, is significantly suppressed because of the long-term assumption of immunosuppressive drugs. In the first reported renal transplant recipient with COVID-19 pneumonia described in Wuhan (China) [129], the clinical characteristics were similar to those of other non-transplanted adult patients with COVID-19 pneumonia; at the end, the patient successfully recovered.

However, kidney-transplant recipients appear to be at particularly high risk for critical COVID-19 illness because of both their chronic immunosuppression and coexisting conditions [130]. Recently, at Montefiore Medical Center, NY, 36 consecutive adult kidney-transplant recipients who were positive for COVID-19 were identified [131], and Columbia University Kidney transplant program enrolled 15 kidney transplant recipients who required hospitalization for confirmed COVID-19 infection [132]. Both reports described management, clinical course and outcomes of those patients living with a kidney transplant affected by COVID-19. Patients were predominantly men, and the median age was 55 years. The most common comorbidities were the following: hypertension, diabetes mellitus, history of smoking and heart disease. Almost all patients were receiving tacrolimus, prednisone and mycophenolate mofetil or mycophenolic acid. The patients reported symptom onset ranging from 1 day to nearly 3 weeks before admission. The most common initial symptom was fever, but also cough, dyspnea, malaise, diarrhea and myalgias. Over 50% of the patients had bilateral/multifocal opacities noted on initial chest x-ray radiographic. Laboratory findings were lymphopenia, thrombocytopenia, low CD3, CD4 and CD8 cell counts. As inflammatory markers, ferritin levels, C- reactive protein, procalcitonin and D-dimer were high. Patients who were in a stable condition without major respiratory symptoms were monitored at home. In another report, among 41 outpatient kidney transplant recipients with suspected or known COVID-19 infection, about one third required hospitalization by the end of their follow-up; there were no differences in demographics or medical comorbidities between those who were or were not admitted to the hospital. These patients therefore required close clinical monitoring to prevent their organ deterioration until symptoms’ resolution [133]. As reported by Montefiore Medical Center and Columbia University, almost half of the patients had AKI, although none had a kidney biopsy performed to determine the cause, and required intubation and mechanical ventilation between 0 and 9 days after admission. In particular, Columbia University’s report observed that 27% of their cases required intubation, a proportion that is similar for cases in New York City overall [132]. Among the patients who developed AKI, only 20% of patients required RRT. At a median follow-up of 21 days, the mortality rate was about 28% of the kidney-transplant recipients [131,132]. Patients were managed with immunosuppression reduction and the addition of hydroxychloroquine and azithromycin.

Therefore, among kidney transplant recipients, overall presentation was similar to the one reported for the general population and more than 50% of the patients were successfully discharged home by the end of follow-up [131]. Regarding treatment of transplant recipients, one challenge was adjusting immunosuppressive agents and at the same time protecting graft function. When treating pneumonia due to opportunistic virus infections following kidney transplantation, a reduction (or even temporary) discontinuation of immunosuppressant drugs is a common therapeutic strategy, consequently allowing recipients the opportunity to reacquire anti-infection immunity within a short period, which is conducive to eliminating the virus [134]. The Columbia University Kidney Transplant Program’s clinical practice has suggested to delay reintroduction of these drugs for up to 2 weeks after discharge, recognizing that prolonged reduction of immunosuppression therapy increases the risk of allograft rejection [132].

Similarly, Alberici et al., in a single center observational study which was conducted in Italy, described a rapid clinical deterioration associated with chest radiographic deterioration and escalating oxygen requirement in 20 kidney transplant recipients with SARS-CoV-2 pneumonia. Thus, in this limited cohort of long-term renal transplant patients, SARS-CoV-2 induced pneumonia is characterized by a high risk of renal progression and a significant mortality rate. Despite on average a relatively benign onset of the disease, a large rate of the patients showed worsening chest radiographs and consequently needed an escalation of the supplemental oxygen. Of note, 25% of the patients died despite an aggressive approach to immunosuppression withdrawal and early administration of antiviral therapy [130].

## 8. Management of the Nephropathic Patient

The patient with chronic kidney disease (CKD) is a fragile patient who in most cases suffers from several comorbidities that predispose to the development of infections [135]. For this reason, after the declaration of the pandemic state by the WHO, protective measures were developed for the patient with CKD without signs of ongoing acute pathology. All nephrology units have been invited to establish a telemedicine system, through which the clinical progress of the patients may be followed. In this way, hospital access was limited only for urgent cases [136].

For patients with the need to prepare arterial venous fistula for the initiation of hemodialysis treatment, the centers specializing in vascular access management organized screening of all patients for COVID-19. Interventions for patients with confirmed or suspected infection needed to be carried out in designated premises with the necessary protection for medical staff [137].

In addition, for kidney transplants, it was necessary to develop preventive measures that could allow for their management. In particular, each Transplant Center had to evaluate the possibility of performing a kidney transplant in relation to the availability of intensive care units and the risk of ICU admission for the individual patient [138]. In addition, during the course of the COVID-19 pandemic, organ donation and transplantation experienced significant changes: organ authorization decreased by 11%; the number of organs transplanted decreased by 18%; and organ recovery for transplantation fell by 17% [139].

Dialysis patients are even more prone to develop severe infectious diseases than the general population [135]. In fact, in previous epidemics or catastrophic situations, the case fatality rate has always been much higher in dialysis patients than in the general population [137]. In addition, for SARS-CoV-2 infection, patients on hemodialysis treatment have proved to be a population at risk of developing COVID-19. In most cases, in fact, they are patients of advanced age and with many comorbidities (cardiovascular disease, high blood pressure, diabetes and lung disease) often associated with worse prognosis in COVID-19 patients [140,141]. The logistical aspects associated with dialysis treatment further increase the risk of contraction and transmission of the disease. The patient on hemodialysis, in fact, must carry out the treatment generally three times a week and this implies the recurrent access to health structures. Furthermore, the hemodialysis session is performed simultaneously by several patients who inevitably interact with each other [142]. These circumstances, associated with the possibility of non-specific clinical manifestations due to immune suppression related to uremia, cause a higher risk of diffusing the infection in the dialysis ward [83].

In some guidance documents, the possibility of performing home hemodialysis with telematic support of the patient and home visits when necessary was also considered [11]. In other papers, however, the reduction of hemodialysis sessions from 3 to 2 times a week has been indicated as an alternative in patients who tolerate this regimen. This would reduce the risk of exposure to infections during transport and the probability of spreading to the dialysis unit or hospital [12].

Being prepared for a sudden and high amount of patients, suspected or confirmed, was crucial for the control of COVID-19 infection.

The Chinese Society of Nephrology [143], the Centers for Disease Control and Prevention, and also the various dialysis organizations developed guidelines for dialysis units during the COVID-19 epidemic. In addition, the EUDIAL ERA-EDTA working group [137] provided recommendations for the prevention and containment of the emerging SARS-CoV-2 pandemic (COVID-19) in hemodialysis centers.

The first step in managing the COVID-19 pandemic in dialysis centers was to ensure training for all healthcare staff. Doctors, nurses and care-workers have been adequately trained on the prevention measures, on the use and disposal of medical devices and any contaminated clothing/objects [137].

In all the centers, a member of staff was assigned to perform the nasopharynx swabs for COVID-19 polymerase chain reaction. The indicated professional figures were trained on the correct methods of performing the diagnostic test but also on the type of devices needed during the procedure. Each operator, in fact, must be equipped with a protective mask (FFP2) capable of filtering 95% of particulates and aerosols in the inhaled air, glasses, mobcap, disposable surgical blouse and gloves [137].

Furthermore, all eligible healthcare staff were trained to perform an accurate telephone triage. The early recognition of suspect patients allows them to be prepared on their arrival, so that they can direct them towards the most suitable path, avoiding interaction with other patients [136].

The second crucial step concerned the training of patients in the dialysis center. In fact, it was important that each patient drastically reduced interpersonal interactions outside the dialysis center, limiting exits only to travel to and from dialysis facilities, using individual transport to reach the dialysis center, decreasing contact with family members, especially with children [137]. The pediatric population, in fact, can act as a carrier of the disease often without showing symptoms or showing mild symptoms [144].

All patients also received instructions relating to how and when to perform hand hygiene, the use of face masks and the disposal of these and any contaminated objects. Family members living with dialysis patients were also sensitized and prompted to follow precautionary procedures, including measuring body temperature, good personal hygiene, frequent hand washing and prompt reporting of potential infected individuals [137].

Of course, organizational adjustments had to be made in dialysis centers as well. The preventive measures, in fact, must start from the waiting room. All dialysis centers have had to adapt their waiting rooms to ensure a safe first reception for patients. It is in fact essential that patients come together in large rooms that can allow a distance of at least 2 m between the patients, equipped with air conditioning and ventilation systems capable of removing particles and droplets of aerosol from the air, equipped with distributors of hydroalcoholic solutions for the hygiene of the hands [137,142].

Additional preventive measures must be implemented in the transition from the waiting room to the treatment room. All patients must be subjected to temperature measurement and must be encouraged to perform hand hygiene and hygiene of the fistula arm, as well as to practice accurate disinfection of the venipuncture areas [145]. At the end of these steps, the patient equipped with a mask can access the treatment room. These, like the waiting rooms, must be equipped with suitable air conditioning/ventilation systems and must guarantee a distance of at least 2 m between the individual positions [137]. 

If patients with suspected symptoms for COVID-19 (fever, cough, conjunctivitis) are identified after the arrival at the Dialysis Center, these must be immediately isolated and subjected to screening tests. Pending the test results, if hemodialysis treatment is not postponed, the patient should be treated as a confirmed case [145]. Therefore, the patient must dialyze in a default room; the room reserved for HBV positive patients could be adequate if available [142]. If the Center does not have a default room, the patient must wait in an isolated room and receive dialysis treatment at the end of the last shift of the day. If, at the end of the treatment, the result of the swab is not present yet, it is necessary to proceed with the immediate disinfection of the rooms [137]. If the outcome of the swab is negative, patients can return to the dialysis facility [145].

The management of patients on dialysis with confirmed COVID-19 infection must be carried out according to strict protocols in order to minimize the risk for both other patients and personnel taking care of these patients. 

Most of the guidelines suggest that dialysis patients, a cause of their management complexity, their fragility and their risk of sudden decompensating, whenever possible, should be hospitalized until complete recovery [145]. The hemodialysis sessions, in this case, can be regularly performed in rooms in isolation or, alternatively, at the patient’s bed with machines usually used for patients with acute renal failure or home hemodialysis machines [136].

In the case of an infected patient with mild symptoms and not hospitalized, the hemodialysis sessions must be performed as his therapeutic scheme, in an isolated environment with appropriately organized medical staff [146]. 

All personnel involved in the direct care of COVID-19 patients must undertake full protection, including hair caps, gloves, long-sleeved waterproof isolation clothing, goggles and medical masks (FFP2 or FFP3 mask, if available) filtering 95–99% of particulate matter and aerosols in inhaled air [137,147].

It is important not only to avoid contact of these patients with healthy patients, but also to prevent the mixing of suspected and confirmed cases [137].

If there are more infected, asymptomatic or paucisymptomatic patients in the same Center, without the need for hospitalization, they can be dialyzed together in a specific section of the unit, preferably as the last shift of the day. A select group of healthcare professionals must be assigned to these patients and must apply all precautionary measures. The hemodialysis room must be properly sanitized at the end of the session and medical waste must be considered highly infectious and disposed of accordingly [137,142].

In case of peripheral centers without the possibility of creating conditions for isolation, and where an infectious disease unit and intensive care unit are both not available, patients with suspected or confirmed COVID-19 disease should be moved into hospitals having these facilities [145].

Management of the peritoneal dialysis patient was less complex than that of the hemodialysis patient. In fact, they usually carry out purification treatment at their home and go to the nephrological center only for scheduled periodic checks. The pandemic from COVID-19 has made the expansion of a telemedicine network necessary to manage patients from home [124].

Telematic support was also indispensable for the management of the transplanted patient. Kidney transplant patients need to perform periodic checks at the post-transplant surgery of the nephrological structure of territorial jurisdiction and at the post-transplant surgery of the Center where the transplant was performed. Telemedicine techniques have made it possible to monitor patients remotely and reduce outpatient visits [136]. 

Finally, in many hospitals the ordinary nephrological hospitalization wards have been closed due to the need to make space for the “COVID areas”. The setting up of a single room for patients awaiting the result of the screening buffer for COVID-19, was fundamental for the restart of admissions for nephrology patients requiring hospital treatment [136].

It is important to emphasize that current guidelines and recommendations relating to the management of renal patients must be considered “provisional” and must undergo continuous reviews and updates in light of new developments.

### Overview of Therapy

COVID-19 treatment is essentially supportive and, in fact, specific vaccines or therapies are not yet available. The COVID-19 vaccine is considered to be an effective prophylactic strategy. About 90 institutions around the world are working on identifying a specific and safe vaccine [148]. As of 8 April 2020, the global research and development landscape for COVID-19 vaccines has included 115 vaccine candidates. Most of these are currently in the preclinical phase and only some of these (mRNA-1273 from Moderna, Ad5-nCoV from CanSino Biologicals, INO-4800 from Inovio and LV-SMENP-DC and pathogen-specific aAPC from Shenzhen Geno-Immune Medical Institute) have recently moved into clinical development [149].

The therapeutic protocols used for the general population have also been adopted in patients with chronic renal failure, regardless of the stage of the disease [150].

Antiviral drugs (Lopinavir/ritonavir (200/50 mg 2 cp × 2/day), Darunavir (800 mg/day), Ritonavir (100 mg/day), Darunavir/cobicistat (800/150 mg/day)) have been effective for the management of SARS-CoV-2 infection, in asymptomatic/paucisymptomatic patients and in patients with more severe manifestations [150,151]. 

Hydroxychloroquine (known as an antimalarial drug) was successfully used in the early stages of the pandemic until May 26th, 2020, when AIFA suspended authorization for its use for the treatment of SARS-CoV-2 infection [152,153].

Tocilizumab (anti-IL-6 monoclonal antibody) has been used in association with steroids and/or antiviral drugs, for the treatment of more severe clinical cases and patients with rapidly and significantly increasing levels of D-dimer. The dosage is 8 mg/kg body weight with a maximum infusion dose of 800 mg for a maximum of three administrations [154,155].

Unlike hydroxychloroquine, both antiviral drugs and tocilizumab do not require dosage adjustments in relation to the values of the glomerular filtrate [150].

Given the state of hypercoagulability and the high incidence of thromboembolic complications in COVID-19 patients, high doses of low molecular weight heparin have been added to the therapeutic protocols [156].

For kidney transplant patients, immunosuppressive therapy must be reshaped, in particular, mycophenolate, azathioprine and calcineurin inhibitors must be suspended during the active phases of the disease [150]. Tocilizumab, on the other hand, may be effective in these patients, but randomized trials are needed [157].

For COVID-19 patients undergoing intermittent dialysis, the methods that obtain the greatest clearance of the pro-inflammatory molecules are preferred; in patients with AKI, hemodynamically unstable and requiring dialysis, continuous dialysis treatments are preferred (CVVH with pre and post-dilution at a dose > 25 mL/Kg/h), using citrate as the first choice anticoagulant (after serial assessments of serum calcium and lactic acid) and, alternatively, unfractionated heparin (constantly monitoring the patient’s APPT) [150].

Interferons have also been proposed as a possible therapy, although the timing of their use is critical since their benefit is realized if given before or early on in infection and may actually be harmful if given in the later stages. Clinical studies are underway to test their efficacy [158].

A recent phosphoproteomics analysis of SARS-CoV-2 infected Vero E6 cells (a cell line derived from monkey kidney epithelial cells) showed dramatic changes in phosphorylation of both host and viral proteins. Furthermore, viral infection induced the formation of casein kinase II (CK2)-containing filopodia containing budding viral particles. The authors have stated that pharmacologic inhibition of several kinases including CK2 and p38 MAP (mitogen-activated protein) kinases may be potential future COVID-19 therapies [159].

A summary of the main contents of this paper is depicted in Table 1.

## Figures and Tables

**Table 1:** Summary of the main evidence emerging from the review.

-	Key-Messages
Epidemiology	The most common chronic preexisting diseases in deceased COVID-19 patients were diabetes mellitus, COPD, arterial hypertension, atrial fibrillation, chronic kidney disease (CKD), active cancer within the previous 5 years, ischemic heart disease and obesity [19]. CKD was present in more than 20% of the deceased patients due to COVID-19 [20].
Pathophysiology	SARS-CoV-2 invades the target cells through a multi-step process that involves cell internalization through interaction with the ACE-2 receptor and priming by the cell protease TMPRSS2 [35,62]. The spike glycoprotein receptor binding domain (RBD) has biomolecular characteristics that are probably responsible for the greater diffusion of SARS-CoV-2 [42]. The renal damage observed in COVID-19 patients is the result of complex mechanisms induced directly and indirectly by SARS-CoV-2 [35]. The two main pathophysiological mechanisms of kidney damage are direct cytopathic effect of SARS-CoV-2 on renal epithelial cells and cytokine storm syndrome [64,71]. Hypoxia, persistent hypotension, rhabdomyolysis, over activation of the coagulation cascade and impairment of microcirculation play a role in the predisposition to the development of acute renal damage [64].
Histopathology	SARS-CoV-2 infection induces acute tubular necrosis through direct cytotoxicity and in immune mediated damage [62,63]. Light microscopy: acute renal proximal tubule injury. Immunohistochemistry: viral nucleocapsid protein into the renal tubular cells of the infected tissues. Transmission electron microscopy: viral inclusion bodies in a peritubular space and viral particles in endothelial cells of the glomerular capillary loops.
Clinical Features	COVID-19 patients with elevated baseline serum creatinine are more likely to be admitted to the intensive care unit and to undergo mechanical ventilation [106]. Kidney disease on admission is a higher risk of negative prognosis [106]. COVID-19 patients with AKI had ~5.3 times mortality risk higher than those without AKI [25]. The incidence of AKI in the overall cohort was in a range of 4.7–7.5% [8,15]. Dialysis patients have a higher rate of contracting SARS compared with the one of the general population, but both the degree of disease severity and the mortality rate of the dialysis group were similar to the general population [113]. SARS-CoV-2 was detected in peritoneal fluid; peritoneal dialysis patients have a higher mortality rate than the general population [119]. Kidney transplant patients have a higher mortality rate than the general population; the clinical manifestations were similar to those reported for the general population, more than half of the patients were successfully discharged home [119].
Management	The diffusion of hygienic-behavioral rules among nephropathic patients and the preventive measures implemented in the various nephrological centers have proven effective in containing the diffusion of COVID-19 among patients with chronic kidney disease (on dialysis and non-dialysis) and transplanted kidney patients [137]. There is no vaccine and therapy specific for COVID-19. Most of vaccine candidates are still in the preclinical phase [149]. Chronic renal failure patients receive the same therapeutic protocols as the general population; these include antiviral drugs, tocilizumab, low molecular weight heparin, hydroxychloroquine (recently stopped by AIFA) [150]. Interferons have also been proposed as a possible therapy [158].
